# The Musculoskeletal Manifestations of Marfan Syndrome: Diagnosis, Impact, and Management

**DOI:** 10.1007/s11926-021-01045-3

**Published:** 2021-11-26

**Authors:** Lily Pollock, Ashley Ridout, James Teh, Colin Nnadi, Dionisios Stavroulias, Alex Pitcher, Edward Blair, Paul Wordsworth, Tonia L. Vincent

**Affiliations:** 1 Kennedy Institute of Rheumatology, NDORMS, University of Oxford, Oxford, UK; 2 Department of Rheumatology, Oxford University Hospitals NHS Foundation Trust, Oxford, UK; 3 Department of Radiology, Oxford University Hospitals NHS Foundation Trust, Oxford, England UK; 4 Department of Surgery, Oxford University Hospitals NHS Foundation Trust, Oxford, UK; 5 Department of Cardiology, Oxford University Hospitals NHS Foundation Trust, Oxford, UK; 6 Department of Clinical Genetics, Oxford University Hospitals NHS Foundation Trust, Oxford, UK

**Keywords:** Marfan syndrome, Scoliosis, Dural ectasia, Pectus deformity, Arachnodactyly

## Abstract

**Purpose of Review:**

Marfan syndrome (MFS) is an autosomal dominant heritable disorder of fibrillin-1 (FBN1) with predominantly ocular, cardiovascular, and musculoskeletal manifestations that has a population prevalence of approximately 1 in 5–10,000 (Chiu et al. Mayo Clin Proc. 89(1):34–42, 146, Dietz 3, Loeys et al. J Med Genet. 47(7):476–85, 4).

**Recent Findings:**

The vascular complications of MFS still pose the greatest threat, but effective management options, such as regular cardiac monitoring and elective surgical intervention, have reduced the risk of life-threatening cardiovascular events, such as aortic dissection. Although cardiovascular morbidity and mortality remains high, these improvements in cardiovascular management have extended the life expectancy of those with MFS by perhaps 30–50 years from an estimated mean of 32 years in 1972 (Dietz 3, Gott et al. Eur J Cardio-thoracic Surg. 10(3):149–58, 147, Murdoch et al. N Engl J Med. 286(15):804–8, 148). The musculoskeletal manifestations of MFS, which to date have received less attention, can also have a significant impact on the quality of life and are likely to become more important as the age of the Marfan syndrome population increases (Hasan et al. Int J Clin Pract. 61(8):1308–1320, 127). In addition, musculoskeletal manifestations are often critically important in the diagnosis of MFS.

**Summary:**

Here, we review the main clinically relevant and diagnostically useful musculoskeletal features of MFS, which together contribute to the “systemic features score” (referred to hereafter as systemic score), part of the revised Ghent nosology for MFS. We discuss current treatment strategies and highlight the need for a multidisciplinary approach to diagnosis and management. Finally, we review new pharmacological approaches that may be disease modifying and could help to improve the outcome for individuals with this syndrome.

## Introduction

**Fig. 1:**
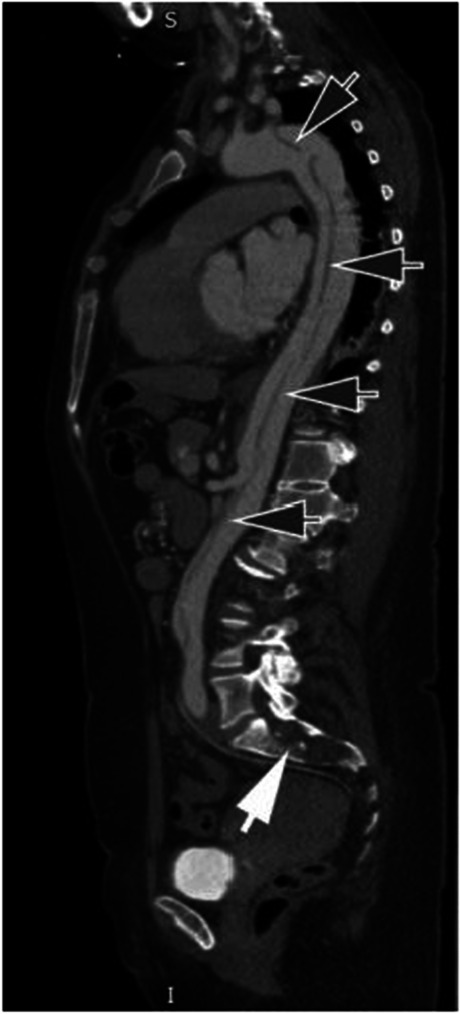
Sagittal CT scan demonstrating extensive aortic dissection (dark arrows) alongside dural ectasia in the sacrum (white arrow)

The criteria for diagnosing Marfan syndrome (MFS) have evolved substantially since the disease was first recognised, in step with our increased understanding of its genetic and pathophysiological features [1, 2]. Making the diagnosis in a timely fashion is essential to ensure that affected individuals are integrated into a regular review to mitigate the risk of acute aortic dissection and progressive cardiac valve disease (Fig. 1). It is also important to distinguish the disease from other disorders with similar manifestations, such as Loeys–Dietz syndrome and vascular (type 4) Ehlers–Danlos syndrome, and also to avoid an incorrect diagnosis in individuals with inconclusive features of MFS [3, 4]. Accordingly, in the most recent revised Ghent nosology in 2010, the importance of cardiovascular manifestations of MFS and the identification of pathogenic mutations in FBN1 are emphasized. Aortic root dilatation and ectopia lentis are the two cardinal clinical features of MFS, but on their own, they are insufficient to confirm the diagnosis. Rather, diagnosis is made using a combination of features stratified on the presence or absence of a family history of MFS (known or presumed), as described in Box 1. This includes the use of a new score for systemic features (systemic score) that include musculoskeletal aspects of MFS, as defined in Box 2. It is important to recognise that the current diagnostic criteria, including the systemic score, were introduced in 2010 so the MFS literature predating this change has become somewhat outdated. Of particular relevance to this review, some of the musculoskeletal criteria that were previously seen as major criteria comparable to aortic aneurysm, such as dural ectasia, are now reduced to a more minor role in the diagnostic process, reflecting their relative non-specificity [4]. Other features that played a role in the earlier diagnostic algorithms, such as high-arched palate and hernias, have disappeared from the list of useful clinical features altogether.

Box 1 Diagnosing Marfan syndrome using the revised Ghent nosology.

**Table:** 

**In the absence of a family history, Marfan syndrome can be diagnosed by:** 1. Aortic dilation (Z score ≥ 2 SD^+^) AND ectopia lentis* 2. Aortic dilation (Z score ≥ 2 SD) AND *FBN1* mutation 3. Aortic dilation (Z score ≥ 2 SD) AND Systemic Score ≥ 7 points* 4. Ectopia lentis AND *FBN1* mutation associated with known aortic dilation **In the presence of a family history of MFS, MFS can be diagnosed by the presence of:** 5. Ectopia lentis 6. Systemic Score ≥ 7 points* 7. Aortic dilation (Z score ≥ 2 SD when above 20 years of age, ≥ 3 SD when below 20 years of age)* ^+^ the Z score is the population score adjusted for surface body area *Caveat: without discriminating features of Shprintzen Goldberg, Loeys-Dietz, or vascular Ehlers-Danlos syndrome **Other related conditions:** **Ectopia lentis syndrome:** isolated ectopia lentis with no *FBN1* mutation or *FBN1* mutation not thought to be associated with aortic dilation **MASS phenotype:** mitral valve prolapse, stable mild aortic dilation (upper limit of normal), skin striae, with at least one skeletal feature without ectopia lentis **Marfan-like body habitus:** Systemic Score ≥ 7 without any other features of MFS	

Box 2 Systemic Features Score—revised Ghent nosology.

**Table:** 

• Wrist AND thumb sign: 3 (wrist OR thumb sign: 1) • Pectus carinatum deformity: 2 (pectus excavatum or chest asymmetry: 1) • Hindfoot deformity: 2 (plain pes planus: 1) • Pneumothorax: 2 • Dural ectasia: 2 • Protrusio acetabuli: 2 • Reduced upper segment/lower segment AND increased arm/height AND no severe scoliosis: 1 • Scoliosis or thoracolumbar kyphosis: 1 • Reduced elbow extension: 1 • Facial features (3/5): 1 (dolichocephaly, enopthalmos, downslanting palpebral fissures, malar hypoplasia, retrognathia) • Skin striae: 1 • Myopia > 3 diopters: 1	

This review comes from the multidisciplinary MFS team in Oxford, run by clinical genetics, cardiology, and rheumatology, and supported by expert teams covering radiology, spinal and cardiothoracic surgery. Collectively, we have experience of over 200 patient families with MFS and see around 100 individuals a year in which a new diagnosis of MFS is being queried. Of these, the majority have a number of musculoskeletal features of MFS (often with a systemic score of around 5); only a small fraction of these will be formally diagnosed with MFS. For those in whom a diagnosis of MFS is made, their care will involve routine echo monitoring (usually yearly) and a review of skeletal manifestations and symptoms over many decades. The purpose of this literature review is to provide a comprehensive and up-to-date portrait of the musculoskeletal aspects of MFS, which contribute to the systemic score. It is hoped that this will be a useful diagnostic and management guide for clinicians. We also highlight clinical manifestations that are likely to become more prevalent as the MFS population ages and identify areas that require further research so that the quality of life of individuals with Marfan syndrome continues to improve.

## Methods

This literature review included papers from January 1960 to March 2020 identified from the PubMed database. Search terms used:

(Marfan syndrome [Title/Abstract]) AND ((facial OR craniofacial) OR (musculoskeletal) OR (chest wall) OR (pectus) OR (scoliosis) OR (hypermobility) OR (protrusio acetabuli) OR (bone mineral density) OR (osteoporosis) OR (dural ectasia) OR (hindfoot valgus) OR (pes planus) OR (osteoarthritis)). Non-English language texts were excluded. A total of 604 were assessed. Abstracts of these papers were screened and case reports excluded. Reviews were assessed separately. Additional papers were retrieved from references therein.

## Hands and Feet

The weight given to deformities of the hands and feet when calculating the systemic score for MFS reflects the importance of these features as a part of the phenotype; in total, 5 points can be scored if all the characteristic signs are observed. In the hand, the wrist and thumb signs complement each other, scoring 3 points when present concurrently, or 1 point if only one is found [5, 6]. In the foot, 2 points are awarded for a hindfoot deformity, specifically “hindfoot valgus in combination with forefoot abduction and lowering of the midfoot”, whilst simple pes planus (flatfoot), considered to be relatively common among the general population, scores only 1 point.

**Fig. 2:**
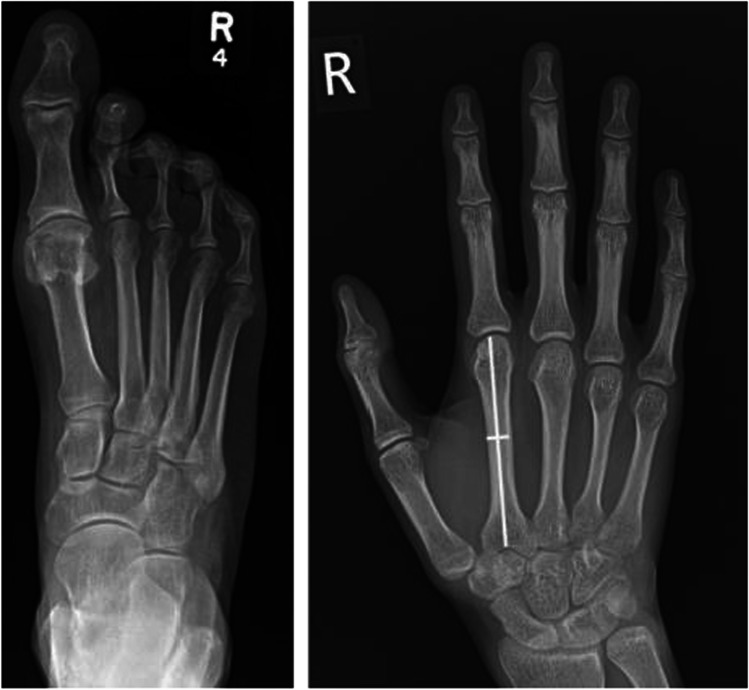
Plain X-rays demonstrating arachnodactyly of the toes and fingers. The metacarpal index is calculated as a ratio of length and width of the metacarpals (white lines) which in this case is 9.5

**Fig. 3:**
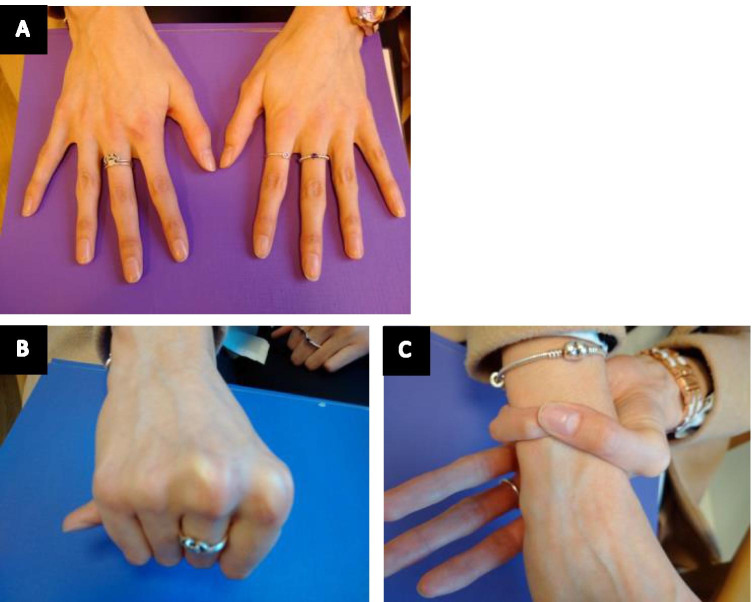
Arachnodactyly in an individual with MFS. Patient with arachnodactyly (**A**). Thumb (Steinberg) sign, with the distal phalanx of the thumb extending beyond the ulnar margin (**B**). Wrist (Walker–Murdoch) sign, with the thumb entirely covering the nail of the little finger (**C**)

Arachnodactyly (which is not included in the Ghent nosology) refers to the long, slender fingers and toes characteristic of the MFS phenotype and can be assessed in multiple ways (Fig. 2). Historically, the metacarpal index (MCI) was calculated from radiographs of the hands to get a sense of the mean length-to-width ratio [7]. However, this ratio was found to be rather nonspecific and become obsolete following the widespread adoption of the wrist and thumb signs, which are far more convenient to evaluate clinically and avoid unnecessary radiation exposure [6, 8, 9]. Arachnodactyly is often easy to spot clinically (Fig. 3A).

The thumb sign, sometimes referred to as the Steinberg sign, is demonstrated when the patient makes a fist over their clenched thumb, and the entire distal phalanx of the thumb protrudes beyond the ulnar border of the palm (with or without the assistance of the examiner) (Fig. 3B) [10, 11]. Despite limited data on the prevalence of this sign among healthy and MFS individuals, it has remained popular due to its simplicity [5, 12]. The same is true of the wrist sign, also known as the Walker–Murdoch sign, where the patient encircles one wrist, proximal to the styloid process, with the thumb and the little finger of the other hand. A positive sign is indicated when digits overlap significantly such that the thumb is able to cover the nail of the little finger completely (Fig. 3C). This reflects both the length of the fingers and narrow wrists, termed dolichostenomelia [5]. Reduced elbow extension is also given one point on the systemic score although, in the authors’ opinion, is not commonly seen in the MFS population.

**Fig. 4:**
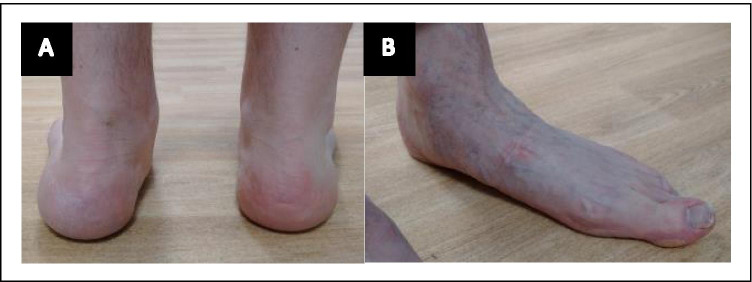
Foot deformity in MFS. **A** Mild valgus hindfoot deformity of left foot (note alignment of heel to Achilles tendon and visible 5th toe). **B** Long slender feet with loss of medial foot arch (flatfeet) in another patient (right image)

The foot deformities seen in MFS, namely hindfoot deformity or simple flatfeet (pes planus) (Fig. 4), have not been as widely studied as other aspects of the disease but can be problematic for patients. An attempt to standardise the definition of pes planus was made by Tareco and colleagues in 1999, based on the percentage force of body weight distribution within the foot. Pes planus was defined by > 24% of body weight transmitted through the medial midfoot, which showed good specificity as only 2.5% of the normal population reached these criteria [13]. By its nature, this is a somewhat arbitrary definition. Nevertheless, it gave an estimate of 25% prevalence among those with MFS, which, more importantly, correlated well with clinical impression on examination.

In 1998, Lindsey et al. investigated foot deformities in those with MFS and found that there was no significant difference in foot function compared with controls [14]. However, individuals with MFS had longer, narrower feet than controls, with more weight being carried through the heel versus the forefoot [14]. The mechanism underlying foot deformity in MFS is often attributed to generalised “ligamentous laxity”, but there is no evidence in the literature to support this; neither of the studies cited above found a relationship between laxity and foot deformities [13, 14]. Aching feet is a relatively common clinical manifestation of foot deformity in MFS, which often responds to orthotics that support the collapsed medial arch. Even when symptoms are not apparent, it is sometimes advised to correct this deformity to prevent symptoms in other joints such as the knees and hips. Toe deformity, such as claw and hammer toes, may occur with MFS and make it difficult for MFS patients to find well-fitting shoes for their long, narrow feet [14].

## Chest Wall Deformity

**Fig. 5:**
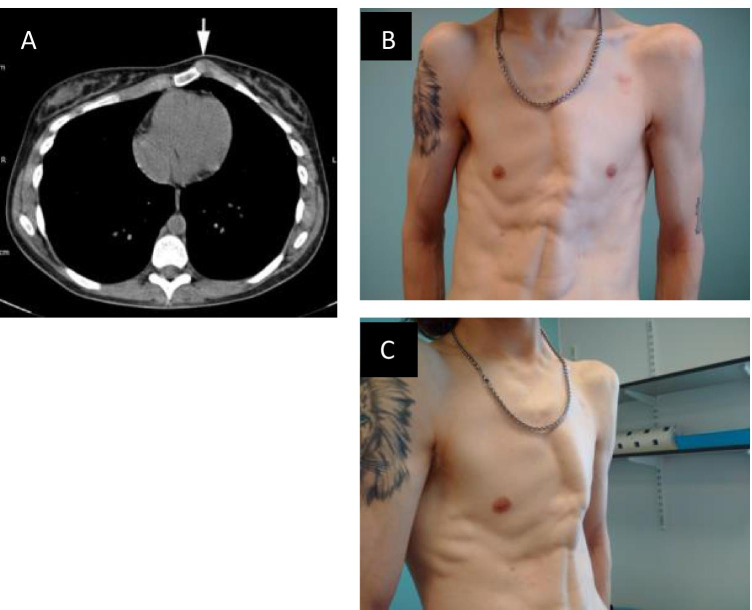
**A** Axial CT scan demonstrating the focal protrusion of the chest wall seen in pectus carinatum (arrow). **B** Frontal view and **C** oblique view of patient with asymmetrical pectus excavatum

Chest wall deformity occurs in up to 70% of cases, usually becoming more evident during periods of accelerated growth in adolescence (Fig. 5) [15–17]. The deformities include either pectus carinatum (PC) (classically a protrusion mimicking the keel of a boat but often referred to as “pigeon chest”) or pectus excavatum (PE) (depression of the sternum, also referred to as funnel chest). Although the majority of the available literature focuses on PE, there is some evidence that PC is the more prevalent chest wall abnormality among MFS patients than PE [4, 16]. Thus, the finding of PC is awarded 2 points whilst PE, or lesser degrees of chest asymmetry, contributes 1 point towards the systemic score (Box 1) [4]. This highlights the fact that PC is thought to be more diagnostically specific for MFS than PE.

The impact of chest wall deformity on cardiovascular and respiratory physiological function has been debated. Clinical symptoms are thought to be related to the severity of the deformity, although even patients with milder deformities may experience persistent symptoms. In particular, PE may cause breathlessness, chest pain (particularly on exertion), and reduced exercise tolerance. These functional changes have been attributed to compressive effects on the mediastinum and consequent impairment of cardiovascular performance [15, 18, 19]. Similar exertion-related symptoms may also occur with PC, probably due to increased chest wall rigidity [17]. In MFS, it is particularly important to establish the underlying cause of such chest symptoms as they could also reflect other associated cardiovascular or respiratory pathologies of the syndrome [15].

**Fig. 6:**
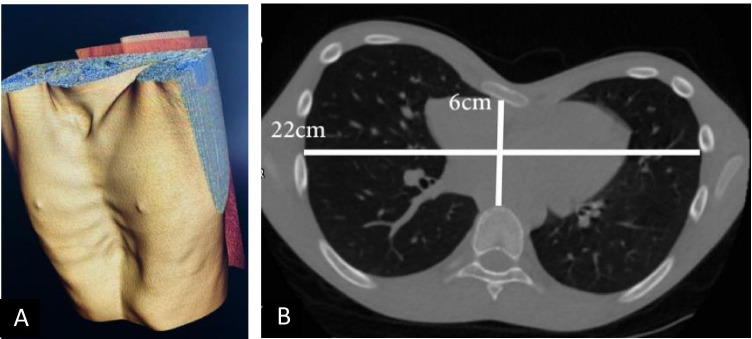
**A** CT reconstruction of thorax from individual with pectus excavatum. **B** The Haller index is calculated by dividing the transverse diameter of the chest by the anterior–posterior distance of the chest on an axial CT slice where the distance between the anterior surface of the vertebral body and the posterior surface of the sternum is the shortest. In this case, the index is 22/6 = 3.67 (severe pectus excavatum). Normal chest: < 2.0, mild excavatum: 2.0–3.2, moderate excavatum: 3.2–3.5, and severe excavatum: > 3.5

There is no consensus on the gold standard thoracic index for PE, but in a survey supervised by the Chest Wall International Group, the Haller index was most commonly utilised. This index can be used to quantify the severity of PE. The index is based on a ratio of the transverse diameter of the inside of the ribcage relative to the anterior–posterior diameter between the deepest part of the sternum and the vertebrae using measurements from CT images (Fig. 6) [20]. However, this index is rarely useful clinically because it varies with age, gender, thoracic shape, and whether the CT is acquired in inspiration or expiration. Furthermore, it does not correlate conclusively with the cosmetic perception of PE. PC does not have such a specific measurement, but anterior–posterior distance may be similarly measured using a simple X-ray or CT (termed HI-Car, Haller index for carinatum).

MRI thorax is increasingly used in the assessment of pectus deformities. Dynamic cine imaging may also add diagnostically useful information in relation to chest wall kinetics as well as delineating the morphology of either PE or PC.

Evidence from a large MFS cohort treated surgically for chest deformities, as reported by Kelly and colleagues and corroborated by other studies, suggests that when PE is present it tends to be more severe than in the general population [21–24]. However, this conclusion is probably biased by the fact that there is a higher threshold for surgical intervention in MFS because of concerns over increased surgical risks and also anxieties over possible complications at the site of surgery should thoracotomy for aortic repair be required in later life.

Even in cases where there is minimal or no physiological effect, chest deformity commonly has a significant psychological impact on affected individuals, resulting in a negative self-image. This impact can be particularly problematic for individuals with PC as it is more difficult to conceal beneath clothing [17, 25]. Patients report teasing and an aversion to undressing in public, which can have a significant impact on their quality of life [25–30]. How MFS patients perceive their chest wall deformity in the context of their cardiovascular disease is an area that is not well addressed in the current literature.

Many MFS patients seek intervention on the basis of a perceived cosmetic defect alone. This is probably the most common reason for treatment, although intervention is also indicated in severe cases that are highly symptomatic (e.g. breathlessness) or where there is an impact on pulmonary and/or cardiovascular function. Factors such as the patient’s age, stage of skeletal maturity height, and suppleness of the anterior chest wall should be taken into consideration when evaluating the available corrective options. There is evidence within the general population for the efficacy of nonsurgical approaches in the treatment of chest wall deformity; these include vacuum bell therapy for pectus excavatum and the use of chest bracing in pectus carinatum [31–35]. Chest bracing has become the mainstay of treatment for pectus carinatum, with operative intervention, such as modified Ravitch procedures [36–38], now rarely undertaken. However, there is little documentation of how effective these treatments are in the long term, specifically for those with MFS.

For excavatum deformities, surgical options include the open Ravitch procedure and the less invasive Nuss procedure, which utilises temporary metal bars, placed beneath the sternum, to restructure the anterior chest wall. A total of 96–100% report a good or excellent result using these procedures [21, 22], although recurrence can occur and may be higher in the MFS population [24, 39]**.** In recent years, recognised good outcome after more conservative measures, such as bracing, means that surgery is largely avoided in the MFS population wherever possible. In the general population, surgical correction of chest wall deformity is justified for purely cosmetic purposes, although NHS England does not currently provide funding for any pectus treatment [28, 40]. Custom-made silicone implants, including breast implants, have also been used for the improvement of the aesthetics of PE. Although this is yet to be implemented or investigated on a wider scale, it could provide a lower risk and less invasive approach [41].

New options in the treatment of PE include the option of combining the repair of pectus deformity and aortic root or arch surgery in MFS patients who require both interventions [39, 42]. These reports caution against concomitant surgery in very young patients, suggesting that the pectus repair should be delayed until the end of the growing period. They also emphasise that pectus deformity in young people may disappear or improve with age as the patient acquires more subcutaneous fat in adulthood and after more conservative management (see above).

## Dural Ectasia

**Fig. 7:**
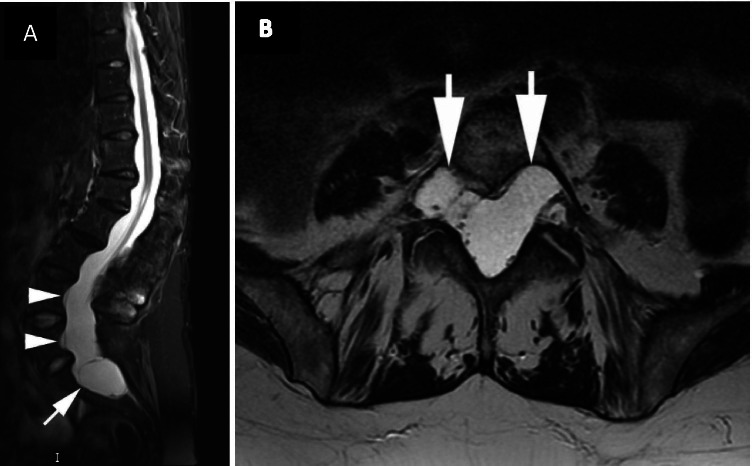
**A** Sagittal short tau inversion recovery (STIR) sequence demonstrating posterior vertebral body scalloping (arrowheads) and severe thinning of the sacrum (long arrow) due to dural ectasia. There is a wider dural sac at the level of S1 compared to L4. Note the kyphoscoliosis of the spine centred at L1–2. **B** Axial T2-weighted MRI demonstrates longstanding scalloping of the posterior aspect of the L5 vertebral body (arrows) due to dural ectasia

Dural ectasia is a widening of the dural sac, most commonly in the lumbosacral spine, with associated changes to the adjacent vertebrae (Fig. 7) [43–45]. Dural ectasia was previously categorised in the Berlin criteria as a major criterion for the diagnosis of MFS, but in the revised Ghent nosology, it was downgraded to a weight of 2 points in the systemic score. It is now believed to be sensitive but not as specific as other major criteria, as it is also sometimes a feature of other connective tissue disorders such as Ehlers–Danlos syndrome [43, 46]. However, dural ectasia is undoubtedly common in MFS with various studies estimating its prevalence between 63 and 97% [46–52].

Both MRI and CT have been used to identify dural ectasia, although MRI is better at visualising the dural sac and hence is generally the preferred imaging modality [48]. Various diagnostic criteria have been proposed, both qualitative and quantitative, and a combination of both approaches is recommended [45, 48, 50, 53–57]. MRI findings include anterior meningoceles, nerve root sleeve dilatation, a wider dural sac at the level of S1 compared to L4, and increased dural sac ratio (DSR), which assesses the relationship between the dural sac diameter (DSD) and the vertebral body diameter (VBD) at the same vertebral level [45].

MFS patients report a number of neurological symptoms that may be related to dural ectasia, including back pain, radicular pain in the thighs and buttocks, genital and rectal pain, headaches, and even neurological deficits [47, 58–60]. However, definitively attributing these symptoms to dural ectasia is complicated by the frequency with which back symptoms occur in the general population. Furthermore, there is conflicting evidence on how these symptoms correlate with the severity of the dural ectasia. Pyeritz et al. reported that even severe dural ectasia may be asymptomatic, whilst Ahn et al. found that there was a significant correlation between increased dural volume and pain [47, 61]. Other reported complications of dural ectasia that deserve further investigation include problems with administering spinal anaesthetics and pooling/dilution of intrathecal drugs due to increased lumbar CSF volume [47, 62].

Two prospective cohort studies have been carried out to date investigating the natural course of dural ectasia in MFS. Mesfin et al. reported in 2013 that over a period of 10.5 years, there was no significant difference in dural ectasia volume, dural sac ratio (dural sac diameter corrected for the size of the vertebral bodies, as defined by Oosterhof et al.), or measured disability in their cohort of 15 patients [57, 63]. In contrast, Boker et al. published a 10-year cohort study of 46 MFS patients that suggested that dural ectasia did worsen with age, although this was a nonsignificant difference [64]. In particular, they found a significant increase in the number of MFS patients developing an anterior meningocele or where the size of meningocele increased. The idea that dural ectasia worsens with age is supported by Sheikhazedah et al.*,* who also reported more marked imaging findings in older patients [56]. If dural ectasia does progress with age, it is important to understand the clinical relevance of this even though there are no specific modifying treatments.

## Protrusio Acetabuli

**Fig. 8:**
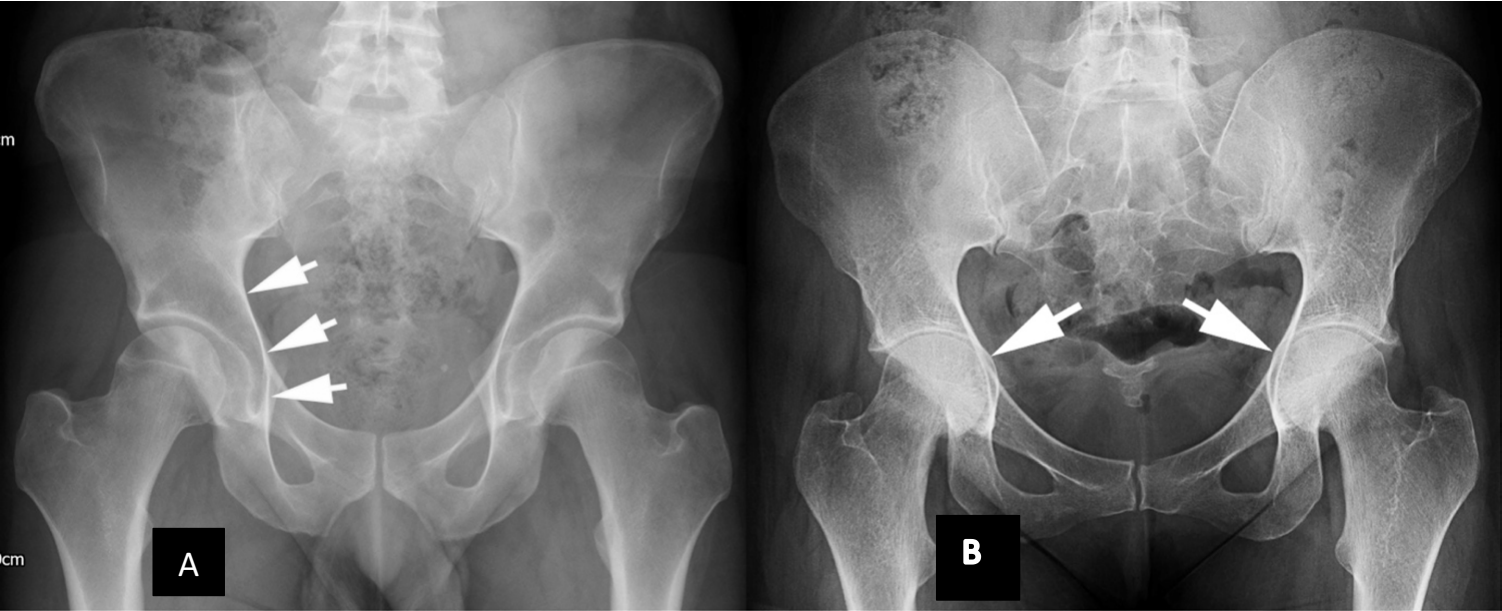
Protrusio acetabuli. **A** Normal appearance. The ilioischial line is shown by the arrows. **B** Protrusio acetabuli. Intrapelvic displacement of the acetabulum and femoral head, with the femoral heads (arrows) projecting medial to the ilioischial line

Protrusio acetabuli is a deformity of the medial wall of the acetabulum that allows the femoral head to migrate progressively towards the pelvic cavity. It is given a weighting of 2 points on the systemic score. Several different definitions and methods of measuring protrusio acetabuli exist (Box 3), which has resulted in a wide range of estimates for its prevalence in MFS, from as low as 16% to as high as 77.4% [65]. This uncertainty makes it difficult to evaluate the impact it has on MFS patients from the available literature [65–69]. In routine clinical practice, a diagnosis of protrusio acetabuli is usually made when the acetabular line is seen to lie medial to the ilioischial line (also called the Kohler line) on plain X-ray imaging (Fig. 8). A number of other methods have been described historically (Box 3).

**Box 3** Previously described methods for assessing protrusio acetabuli.

**Table:** 

•Centre-edge angle of Wiberg (steel) •Kulman •Armbuster Circle-wall distance (Lundby)	

Historically, surgical closure of the triradiate cartilage physis in children with protrusio acetabuli has been performed with the intention of preventing progressive deformity and degenerative change [68]. This surgical procedure was still being performed as recently as 2005 [69, 70]. The need for surgery has been reassessed in recent years, by Sponseller and colleagues [66]. They performed a cross-sectional study involving 173 MFS patients with protrusio acetabuli, diagnosed using both Steel and Armbuster methods for comparison (27% and 16%, respectively) [66, 70, 71]. Crucially, and unlike most other studies, they also investigated the hip symptoms that these patients were experiencing. They failed to find a strong association between the presence of protrusio acetabuli and hip problems; for those with reduced hip function, they proposed that” their hip symptoms were more likely due to an unrelated process, degenerative, or otherwise”. Furthermore, they observed that the incidence of protrusio acetabuli was largely associated with the first 2 decades of life before plateauing in patients in their 20 s, suggesting that progressive deformity may slow as the skeleton matures. There is an absence of longitudinal studies on protrusio acetabuli to date.

Until recently, anteroposterior pelvic radiographs were the only method used for identifying protrusio acetabuli. A new method reported by Lundby et al. utilises CT scanning, where pelvic tilt can be corrected before protrusio assessments were made [72, 73]. This method reveals very high estimates of the frequency of protrusio in MFS patients [67, 72]. Despite the potential benefits, it seems questionable to use CT in the diagnosis of protrusio acetabuli, particularly as the authors acknowledge that it would only have significantly impacted the diagnosis of MFS in one individual included in their study. Further, the use of protrusio as a diagnostic aid in MFS was developed from plain radiographic assessment. Unless there is a clear clinical justification for the additional radiation exposure, it seems questionable to expose young patients to the extra risk for purely diagnostic purposes.

## Scoliosis and Spinal Deformity

**Fig. 9:**
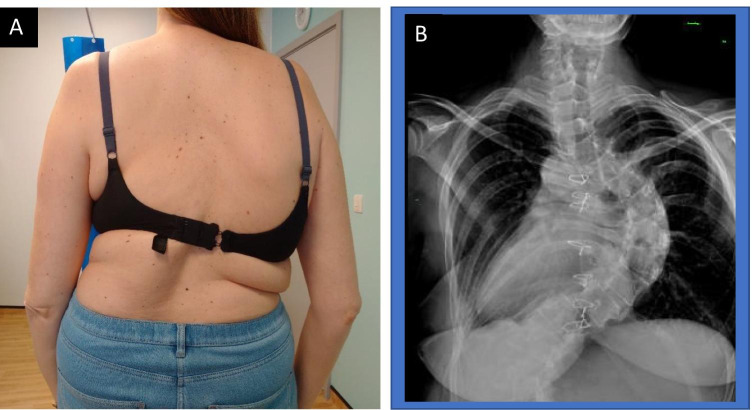
**A** Clinical appearance and **B** X-ray appearance of severe scoliosis in a patient with Marfan syndrome. Marked “double curvature” results in apparent clinical compensation although the length of the thorax (and therefore height) is markedly shortened as a result. This patient had relatively few symptoms despite deformity. Note X-ray reversed to mirror patient position, also evidence of previous sternotomy for cardiac surgery

Scoliosis consists of lateral deviation of the spine combined with axial rotation and vertebral body wedging. It can be one of the more severe and striking skeletal manifestations of MFS, but it contributes only one point to the systemic score, probably because idiopathic scoliosis is relatively common in the non-MFS population (Fig. 9) [74]. Thoracolumbar kyphosis may also be a spinal manifestation and also scores one point. Scoliosis affects more than 50% of MFS patients, as defined using the Cobb method that considers an angle of > 10° significant [75, 76]. However, the 2010 Ghent nosology suggests that scoliosis in MFS should be defined as an angle of > 20° to increase specificity. When comparing the scoliosis of MFS to adolescent idiopathic scoliosis (AIS) in the general population, there are several significant differences to be noted. In particular, there is an earlier age of onset in MFS patients, which may relate to an increased severity of the deformity. There are also differences in the curve pattern in MFS patients, with a higher rate of double thoracic and triple major curves [77–79]. Additionally, there is an increased tendency for hyperkyphosis (> 50°) in MFS, whereas in AIS the scoliosis is more commonly accompanied by hypokyphosis. AIS is more common in females, whereas scoliosis in MFS patients has an equal sex ratio.

These differences in the type of scoliosis may reflect more general variations in the morphology of the spine in individuals with MFS. In particular, there is evidence that individuals with MFS have an increased number of transitional vertebrae, reduced pedicle width, reduced laminar thickness, and increased interpediculate distances [75, 80, 81]. These, and other, differences in the lumbar spine may directly relate to the co-occurrence of dural ectasia in MFS, which impacts the osseous anatomy (see below). An increase in mobility of the lumbar spine due to ligamentous laxity has been postulated, but there is limited evidence for this in MFS compared with the general population [82]. In a large study of MFS, abnormalities of the cervical spine (focal kyphosis, basilar impression, and increased mobility) appear to be more common but are not associated with notable clinical problems or a significant increase in neck pain [83].

The treatment of scoliosis in Marfan syndrome has been discussed widely in the literature, reflecting the fact that this skeletal problem remains a major challenge. In AIS patients, bracing is often used with good results when used in skeletally immature patients with curves between 20 and 40°. In these cases, 60–80% of curves will not progress more than 5° [79, 84–86]. However, similar success was not achieved in MFS patients as described in a comprehensive retrospective review of scoliosis by Sponseller and colleagues (2000). In a cohort of patients meeting the same spinal criteria as in AIS, only 17% of MFS patients achieved progression rates of less than 5°, even with near full-time brace wear [79]. These relatively poor outcomes are corroborated by other studies. Suggested factors that may contribute to the failure of bracing include altered force transmission through ribs to spine, increased rigidity of the curve, earlier age of scoliosis onset, and reduced tolerance for bracing in MFS patients (thinner subcutaneous layer and potential cardiopulmonary compromise) [77–79].

**Fig. 10:**
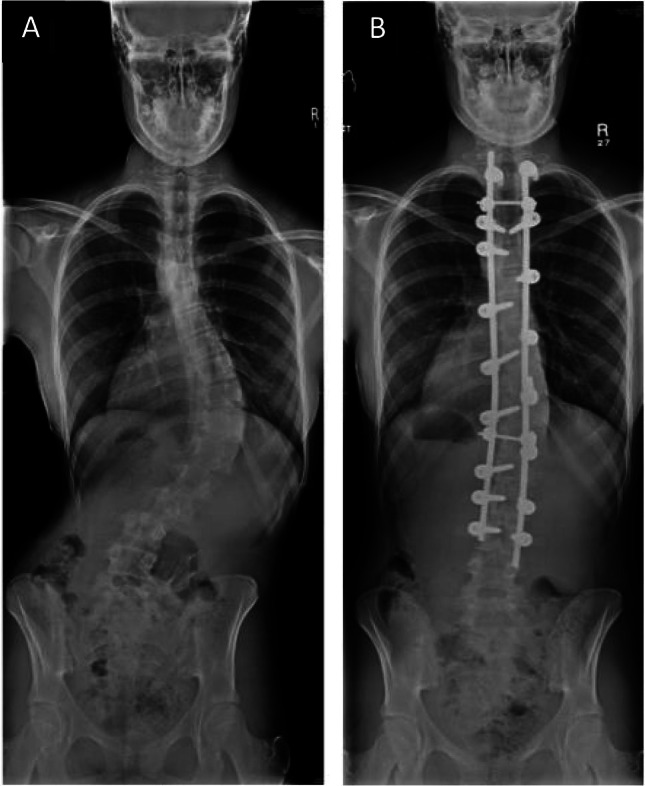
X-ray appearance pre (**A**) and post (**B**) surgical correction of scoliosis in individual with MFS

When bracing is unsuccessful, or the curve is regarded as too severe to brace, surgery may be needed to control progressive deformity (Fig. 10). This usually consists of spinal fusion, with an increasing preference for posterior-only fusions, which are usually as efficacious as anterior release combined with posterior spinal fusion and have the added benefit of reduced blood loss and operation time [87–90]. There is conflicting evidence in the literature over whether there is a higher rate of complications associated with spinal fusion in MFS compared with AIS, but the general view would be that these constitute a high-risk category of patients. Most studies have found that there is greater blood loss in MFS patients compared with those with AIS, a higher rate of intraoperative CSF leaks (likely due to dural ectasia), and of decompensation and curve progression [90–93]. It has been suggested that the risk of the latter can be minimised if there is careful planning of the fusion levels, often requiring a longer fusion including neutral vertebrae above and below the curve, and avoidance of initial overcorrection of the curve [87, 91, 92, 94]. Aggressive pre-operative optimisation of cardiac comorbidities and perioperative management of possible anticoagulation therapy is necessary. Bone density is reduced in MFS, which may influence the choice of implants and can lead to fixation failure later on. Intraoperatively, consideration should be given to positioning the patient in the Trendelenburg position to reduce dural pressure and lessen the risk of dural tears.

## Craniofacial

Facial features considered characteristic of MFS are also included when calculating the systemic score. These are dolichocephaly (relatively long skull), enophthalmos (abnormally retro-positioned globe with respect to the bony orbit), down-slanting palpebral fissures (the external palpebral fissure sits below the level of the internal one), malar hypoplasia (underdevelopment of the cheek bone), and retrognathia (lower jaw is set further back than the upper jaw); a patient must exhibit at least three of the five features listed in order to score 1 point. In a case–control study, retrognathia and down-slanting palpebral fissures were the most prevalent facial features in the MFS population, and this is supported by other studies [95–97].

The clinical utility of facial features in the detection of MFS is naturally dependent on the experience of the examining clinician. Ting et al. (2010) reported that experienced clinicians can discriminate between patients and controls with an overall accuracy rate of ~ 70%, but those with limited clinical exposure to MFS are unlikely to reach the same precision [98]. In the same study, variation in facial features with age was also noted, and this affected the clinician’s ability to recognise MFS. Clinicians demonstrated a higher accuracy, sensitivity, and specificity in the 0–10 years age group, pointing to a greater clinical utility for discerning facial features in children.

Recent research has focused on how advances in 3D facial analysis technology may help to further characterise the facial features in MFS and, in time, increase their clinical utility in diagnosis. For example, digital stereophotogrammetric techniques map soft-tissue landmarks of the face for measuring linear distances and angles between these points, which can be used to calculate *z*-scores for comparing MFS patients to a reference group [99–101]. Those with MFS have a significantly reduced facial divergence (midfacial to mandibular plane angle), which will be altered in those with retrognathism. They also may have a reduced facial height index, contributing to dolichocephaly. These features have not been specifically compared with those mentioned in the Ghent nosology. This technique has the benefit of not using radiation, unlike previous cephalometric X-ray-based studies looking at changes in the craniofacial skeleton [96, 97].

It is important to recognise that other craniofacial features that are excluded from the systemic score are also regularly reported in MFS; notably, orthodontic changes such as the high arched and narrow palate [96, 102]. Despite evidence that a high-arched palate and associated dental crowding is present in a large proportion of MFS patients, this feature was removed from the current nosology “because of lack of perceived specificity” [4, 96, 103]. Although no longer a formal contributor to the systemic score, when combined with other reported orthodontic features such as cross-bite, Class II molar occlusion, and potential increased risk of dental caries, it appears that oral manifestations of MFS are often identifiable on routine examination [95, 104, 105]. An awareness of these features among dental practitioners and orthodontists is helpful for raising the suspicion of MFS due to the risk of bacterial endocarditis in those with heart valve involvement, which may warrant antibiotic prophylaxis [3, 105, 106].

Other craniofacial features of MFS are also mentioned in the literature. They include increased nasal airway resistance, likely due to constriction of the maxillary arch, which may contribute to sleep apnoea in certain patients. MFS patients also have an increased prevalence of temporomandibular joint disorders [107, 108].

## Bone Mineral Density and Fracture Risk

The relationship between MFS and bone mineral density (BMD) has long been a source of contention; generalised osteopenia was first described in 1990 by Magid et al., but subsequent studies have produced conflicting results [109–119]. Overall, there appears to be some evidence for reduced BMD in MFS patients, but the clinical significance of this change has not been fully elucidated.

In studies that have demonstrated a reduced BMD in MFS, evidence comes from the assessment of sites including the wrist, hip, femoral neck, and lumbar spine. There is debate over the utility of assessing the lumbar spine due to the potential confounding effects of concurrent scoliosis or dural ectasia [110–116]. Patient characteristics vary widely between the groups that have been studied, although gender is not a source of conflicting results as both male and female patients seem to be affected by reduced BMD [112, 113]. Several studies have also focused specifically on BMD in children, using correction techniques to interpret the DXA scans. These studies provide some evidence that a reduced BMD may be present from a young age [111, 115, 116, 120]. However, measurement of BMD using DXA scanning in children must be approached and interpreted with particular caution due to many potentially confounding effects [121]**.** There are currently no longitudinal studies examining BMD with age in MFS [102].

Some of the conflicting evidence regarding BMD has been attributed to differences in the methods and techniques used. In 2003, Giampietro and colleagues proposed that the studies using one type of DXA machine (Hologic instrumentation) produced results showing reduced BMD compared to reference ranges, whereas those using another (Lunar DXPL instrumentation) detected no difference [119]. Studies performed by Trifiro et al. (2015) using the Hologic DXA measurement have since also demonstrated a reduction in BMD in a MFS cohort, so whether the instrumentation used to measure BMD has truly affected the studies in question remains uncertain [116]. Further confusion arises from comparisons relying on *z*-scores (the age-adjusted standard deviation defined by a normal population) alone, making it very challenging to compare the results of different groups, as determinants of *z*-scores vary between studies. Ideally, comparisons of *T*-scores (the non-age-adjusted standard deviation where comparison is with a defined peak BMD in a normal population), would overcome this issue in adults, but these are infrequently reported in published studies. *T*-scores would also not be appropriate for children [119].

Critically, relatively few studies have examined whether the putative reduction in BMD predisposes individuals with MFS to a higher fracture risk compared with the general population. One of the earliest studies by Kohlmeier et al. in 1993 found no link between the reduced BMD in MFS and an increased number of fractures. However, in a later study, they suggested that women with MFS may have a 20-fold increased risk of hip fracture as a consequence of both reduced BMD at the hip and an increased hip axis length, which is associated with increased fracture risk [110]. Moura et al. reported significantly reduced BMD at the hip and wrist. In addition, 24.6% of individuals with MFS had a history of trauma-related fracture, although the study lacked data on the prevalence of fracture types within a matched normal population [114]. Most recently Trifiro et al. (2020) published the only study to date on fracture incidence, in this case within the paediatric population [120]. They report a fracture incidence of 29.3/1000 per year in children with MFS compared to 15.8/1000 per year in children without MFS from the same geographical location (*P* = 0.034) [120]. They report that these fractures were predominantly of the upper limb and occurred following low or moderate energy trauma. Despite this apparent increase in fracture rate among children with MFS, they failed to demonstrate any correlation between low BMD and fracture risk. This finding highlights other potential fracture risks associated with MFS such as altered biomechanics of the skeleton due to elongated long bones and altered bone microarchitecture and increased risk of fall because of low muscle bulk, poor visual acuity, and poor exercise tolerance.

More rigorous longitudinal studies are required to establish whether the reduction in BMD and the apparent increase in fracture risk in MFS are clinically important. It will be particularly valuable to perform this in the older MFS population against a background of known increased fracture risk associated with age.

## Patient QoL/Pain

An overlooked but important effect of the musculoskeletal complications of MFS is their impact on daily functioning and quality of life. This aspect is often neglected from studies focusing on specific characteristics of MFS and there is only a handful of studies that take a more holistic view of the experience and perceptions of patients. However, the number of such studies has been increasing in recent years, perhaps reflecting a general shift to a more patient-centred approach.

The impact of MFS on health-related quality of life, as assessed by the 36-item short-form survey (SF-36), has consistently and perhaps unsurprisingly reported reduced scores (poorer QoL) in MFS compared with the population average of 50 [59, 122–124]. However, the correlation between reduced QoL and disease severity or specific features of the condition is less clear-cut. The specific impacts of the musculoskeletal manifestations of MFS on quality of life can be broadly divided into two categories: (1) those that cause pain and that negatively impact daily function and (2) those that lead to poor body image and associated psychological upset.

Pain can be a pervasive and debilitating symptom of MFS, yet it remains poorly defined and often poorly controlled. In Speed’s study in 2017 involving 245 MFS participants, 89% reported pain, most commonly beginning in the back (50.6%). Of these, only 46.6% were satisfied with their current pain management [125]. Symptoms associated with pain included stiffness, difficulty walking, muscle spasms, and muscle weakness, and there was a significant association between pain and the presence of kyphosis, degenerative disc disease, osteoarthritis, and dural ectasia. The presence of chronically sore joints has also been shown to correlate strongly with a negative patient perception of how controllable MFS is as a disease [126]. The source of pain is often ill-defined, and determining how much of the pain experienced by individuals with MFS is specifically attributable to their disease remains challenging, especially when their symptoms are common in the non-MFS population. However, it is important to take symptoms of pain seriously and to investigate and treat them actively. In some individuals, pain may be due to osteoarthritis, chest deformities, nerve compression due to scoliosis, dural ectasia, and bony overgrowth [59, 125, 127–130]. Pain in MFS has also been reported to be more severe in older patients, hence particular care should be taken not to overlook pain management when treating this patient group [125, 131]. Despite having generally low muscle support across joints, an increase of osteoarthritis in individuals with MFS has not been reported. This would be in contrast to that seen after acute joint instability (e.g. after a ligamentous injury) or in some forms of Loeys–Dietz syndrome.

Recent research by Warnink-Kavelaars and colleagues (2019) investigated the impact of MFS on daily functioning, particularly among children and adolescents with MFS [132, 133]. The key theme identified through semi-structured interviews with parents and adolescents related to an inability to keep up with peers, across various aspects of life including at school, work, leisure, and sport, attributed to” fatigue, pain, and physical impairments”.

The diagnosis of a chronic and potentially life-threatening disease such as MFS comes with its own psychological burden that varies widely depending on personal circumstance, particularly when the manifestations and severity of the condition vary widely. An investigation into patient perception of MFS identified negative self-image driven by physical features as a common concern. This is in accordance with the negative psychological impact of chest deformity and scoliosis, previously discussed. However, importantly, not all patients viewed the musculoskeletal manifestations in a negative light. Indeed, there was evidence that many enjoyed the advantages of being naturally tall and slim and individuals over the age of 13 generally had a positive self-image [3, 126, 134].

## Opinion

Only a small fraction of patients referred with possible MFS will be formally diagnosed with MFS. MRI and X-ray imaging to identify dural ectasia and/or protrusio acetabuli may occasionally be of value to increase the systemic score, but these are usually negative. In those individuals with a systemic score greater or equal to 7/20, but with an absence of cardiac or eye involvement or positive family history, it is often judged prudent to repeat echocardiograms (3–5 yearly until around 25 years of age) to ensure that they are not evolving from an “isolated Marfan-like body habitus” to MFS. There are no clear guidelines for these cases and their skeletal features are often relatively mild.

Whilst our experience concurs with much of what is cited above, we also recognise a number of additional common problems that face individuals with MFS. Pain is often a minor symptom in the younger patient but can become increasingly problematic with age. This sometimes presents as part of a wider chronic pain syndrome, often complicated by stress. Pain is also a relatively frequent complaint of our older patients who have had spinal surgery in the past for correction of scoliosis. This is presumed to be due to osteoarthritis developing at the junction between the fused and non-fused vertebrae where additional mechanical stress occurs. Similarly, older patients may struggle with foot pain, as a consequence of mechanical strain due to deformity and poor support over time. Management of these poses significant challenges, as conventional pain relief is often inadequate.

For parents of children with MFS, there are a number of practical issues that they find hard to manage and for which the schools are often ill-equipped to deal with (due to lack of knowledge of the disease). For instance, children often struggle to find school shoes that fit, especially if they also have to accommodate orthotics. Whilst a note to the school to support wearing sneakers may be a solution, children often don’t like to stand out as “different” from their peers. Fatigue is very common in children with MFS, and the school needs to make allowance for this by providing flexibility when partaking in physical education and extra time for activities. All individuals with MFS are discouraged from contact sports (rugby, boxing), heavy weightlifting (that involve the Valsalva manoeuvre), high G-force activities (including extreme fair-ground rides), and deep-sea diving because of the risk of pneumothorax and strain on the vasculature and retina (especially in those with severe myopia who are at risk of retinal detachment). Focus on academic work may be affected by upper limb fatigue when writing and poor visual acuity (when there is lens involvement).

## Future Directions

Research in MFS has expanded rapidly in the past 10 years with new molecular insights suggesting that dysregulation of TGF*β* signalling may be a central mechanism of disease pathogenesis in Marfan syndrome [135]. Treatments with angiotensin antagonists that target TGF*β* signalling as well as control blood pressure have been shown to prevent aortic dilation and the development of mitral valve prolapse in mice with MFS [136, 137]. As a result of these studies, a number of randomised controlled studies have been carried out in individuals with MFS [138–144]. Although some of these failed to meet the primary study endpoint when compared with standard treatment (*β*-blocker), others showed superiority when compared with placebo or when the study was extended beyond the original follow-up period [138, 144, 145]. An ongoing meta-analysis of these studies will help clarify the overall effects of treatments and may identify particular phenotypes (including musculoskeletal) that are most amenable to treatment. It is unclear at this time whether the musculoskeletal manifestations of MFS could be amenable to TGF*β* targeting in the same way.

## Conclusion

The correct identification of physical signs in the musculoskeletal system remains a central element of the assessment of the patient with suspected MFS, and when taken together with an evaluation of other systems, notably the eyes and cardiovascular system, can allow for reliable clinical diagnosis (supported by echocardiography and slit-lamp evaluation) in most cases.

The most important potential consequence of the diagnosis of MFS is the identification of a dramatically increased risk of cardiovascular catastrophe (probably about 250-fold overall, and up to 1000-fold in some age and sex groups), which one seeks to avoid by regular monitoring, medical therapy, and timely surgical intervention for those at the highest risk. The risk is greatly increased when the diameter of the aortic valve ring at the sinus of Valsalva reaches 5 cm or so. Elective surgery to the proximal aorta and/or valve at this point is preferable to enforced surgical treatment as a medical emergency.

The substantial improvements in life expectancy that have been achieved since the early 1970s have had two effects: (1) a greater imperative to diagnose patients at an early stage to allow effective preventative approaches and (2) a longer timescale over which both vascular and nonvascular effects can manifest. Among these nonvascular manifestations are musculoskeletal effects, and the patient who escapes these entirely is rare. A comprehensive approach to the care of patients with MFS will involve periodic (re)assessment of the musculoskeletal aspects of the condition and patients’ needs in respect of them. The lifetime incidence of musculoskeletal complications of MFS has not been ascertained with certainty. Prospective clinical studies of older adults are needed to capture aspects of the condition that develop or worsen over time. Studies are also needed to reflect the diverse ethnic groups affected by MFS and attention should be paid to measures of quality of life and psychological aspects. A multidisciplinary team approach to patient management and enhanced research in this area is called for.

## Data Availability

Not applicable.
